# Thyroid Autoimmunity in Female Infertility and Assisted Reproductive Technology Outcome

**DOI:** 10.3389/fendo.2022.768363

**Published:** 2022-05-26

**Authors:** Ines Bucci, Cesidio Giuliani, Giulia Di Dalmazi, Gloria Formoso, Giorgio Napolitano

**Affiliations:** ^1^Center for Advanced Studies and Technology (CAST), University “G. d’Annunzio” of Chieti‐Pescara, Chieti, Italy; ^2^Department of Medicine and Aging Science, University “G. d’Annunzio” of Chieti‐Pescara, Chieti, Italy

**Keywords:** thyroid autoimmunity, female infertility, assisted conception, assisted reproduction technology, pregnancy outcome, miscarriage, thyroid peroxidase antibodies, thyroglobulin antibodies

## Abstract

The regulation of the female reproductive system is one of the most relevant actions of thyroid hormones. Adequate thyroid hormones production is essential for normal menstrual function and fertility as well as for the successful maintenance of pregnancy. The relationship between reproductive failure and thyroid disorders is particularly relevant and attracts attention worldwide. Thyroid autoimmunity (TAI), defined by the presence of circulating antithyroid antibodies targeting thyroid peroxidase (TPOAb) and thyroglobulin (TgAb), is prevalent among women of reproductive age and is the most frequent cause of thyroid dysfunction. Several studies addressed the association between TAI, thyroid function, and fertility as well as pregnancy outcome after spontaneous or assisted conception. Infertility, miscarriages, and fetal-maternal complications are described in overt autoimmune hypothyroidism. More debatable is the role of mild thyroid dysfunction, mainly subclinical hypothyroidism (SCH), and TAI in the absence of thyroid dysfunction in infertility and reproductive outcome. Assisted reproductive technology (ART) has become an integral element of care for infertility. Women with TAI undergoing ART are of particular interest since they carry a higher risk of developing hypothyroidism after the ovarian stimulation but whether TAI, in absence of thyroid dysfunction, adversely affects ART outcome is still controversial. Likewise, the role of levothyroxine (LT4) in improving fertility and the success of ART in euthyroid women with TAI is unclear. This review discusses the role of TAI, in the absence of thyroid dysfunction, in infertility and in ART outcome.

## Introduction

The definition of infertility is the failure to achieve clinical pregnancy after 12 months or more of regular unprotected sexual intercourse (1). Female factors (ovulatory disorders, endometriosis, pelvic adhesions, tubal blockage/abnormalities, and hyperprolactinemia) account for 35%, male factors for 30%, and combined factors for 20%. In 15% of the cases the cause remains unknown (idiopathic or unexplained infertility). The prevalence of infertility varies worldwide and is estimated to affect between 8 and 12% of reproductive-aged couples (2). Multiple elements can contribute to infertility affecting the female, the male, or both partners and many studies have been dedicated to identifying treatable risk factors that contribute to infertility. Among these risk factors are the presence of thyroid dysfunction and/or TAI. Therefore, screening for TSH and TPOAb is generally part of the initial work-up of infertile women. Indeed, thyroid hormones (TH) regulate the hypothalamic-pituitary-ovarian axis. A role of TH on luteinizing hormone (LH) secretion, on granulosa cell functions, LH/hCG receptor expression, and follicle development has been demonstrated (3). Therefore, ovulatory dysfunction and infertility are common in untreated thyroid dysfunction (4). Moreover, TH plays a critical role in implantation and early fetal development through actions on the placenta and endometrium (5). If adequate TH is necessary to avoid reducing the chances of conception and implantation, the same is true for the maintenance and outcome of pregnancy as well as for fetal brain development. In pregnancy significant adaptations of thyroid function are required, and the maternal thyroid must increase the hormone production by approximately 50% (6, 7). These pregnancy related adaptations cannot be accomplished in women with undiagnosed or uncontrolled hypothyroidism. The adaptation is even more difficult in assisted conception since the controlled ovarian stimulation protocols of ART anticipate the strain on thyroid function observed only after conception in spontaneous pregnancy (8). Thyroid disorders are more prevalent in females and are frequently encountered as a new diagnosis in women seeking pregnancy or during pregnancy. TAI is characterized by the presence of circulating antithyroid antibodies targeting thyroid peroxidase (TPOAb) and thyroglobulin (TgAb). Indeed, also circulating antibodies targeting TSH receptor, that are hallmarks of Graves’ disease, have significant implications in reproductive age women and in pregnancy but their discussion is beyond the scope of this work and has been reviewed elsewhere (9). TAI is the leading cause of thyroid dysfunction and comprises a spectrum of conditions ranging from euthyroid thyroiditis to subclinical or overt hypothyroidism and, less frequently, to transient thyrotoxicosis. There is evidence that TAI-related thyroid dysfunction (mainly overt and subclinical hypothyroidism) adversely affects conception and pregnancy outcomes (10). Rather, it is unclear what impact TAI has with normal thyroid function in infertility, and in the outcome of pregnancy especially in women undergoing ART; clinical studies as well as meta-analyses report discrepant results (11). Despite a multitude of studies, a clear pathophysiological link connecting TAI and reproductive failure has not been identified. It is challenging to distinguish the effect of TAI per se from the changes it can induce in thyroid function, that is, TSH levels at the high end of the normal range or compatible with subclinical and overt hypothyroidism. When looking at studies addressing the role of TAI-related thyroid dysfunction in reproductive failure it must be remembered that the serum TSH level cut-off used to define normal thyroid function in pregnancy has been changed over time. The upper limit of serum TSH was set at 2.5 mIU/L according to the American Thyroid Association (ATA) guidelines published in 2011 (12). In the newest guidelines published in 2017, the need for trimester specific and local population TSH reference is strongly reaffirmed and, if this is not available, the upper limit of TSH in the first trimester is set at 4.0 mIU/L (13). Recently, guidelines specifically addressing thyroid dysfunction prior to or during ART have been published by the European Thyroid Association (ETA) (14). In searching a thyroid function independent pathogenic role of TAI in reproductive failure, several studies have been dedicated to potential immune-dependent direct effect on the ovary, on the endometrium, and on the feto-maternal unit. The findings are interesting but merely speculative and need confirmation (15). There remain unanswered questions on the relationship between TAI and reproductive outcome; several fields of research are open (11, 16). Increasing knowledge on the pathophysiological link between TAI and reproductive failure would improve the management and treatment of infertile women with TAI. The treatment of infertile euthyroid women with TAI remains the greatest areas of uncertainty, some studies demonstrate that LT4 reduces the risk of adverse pregnancy outcome while others do not (17). In this work we reviewed the epidemiological, clinical, and pathophysiological data addressing the relationship of TAI, without thyroid dysfunction, with infertility and ART outcome.

## Prevalence of Thyroid Autoimmunity in Infertile Patients

The prevalence of TAI differs with races, age, iodine supply, and smoking, and is estimated as high as 5–16% in women aged 20–45 years in Europe (18, 19). Many studies have investigated the prevalence of TAI in infertile women. The studies are heterogeneous for design (frequently cross-sectional or retrospective), sample size, subjects (women with different causes of infertility or with a selected cause) and controls (unselected, age matched fertile, healthy non-pregnant) and for different methods of autoantibodies assay. In studies published until the early 2000s the prevalence of TAI in infertile women ranged from 6.8 to 14.5% but without significant differences compared to controls (20, 21). Only in two studies the prevalence was higher than the control population in women with anovulation (26%), in women with idiopathic infertility, and, mostly, with endometriosis (30%) compared to the unselected population (22, 23). However pooling the result of the studies the prevalence of TAI among women attending infertility clinics has been estimated higher compared to the general population with a relative risk of 2.1 (24). In more recent studies a prevalence of TAI in infertile women between 13% and 19% has been reported (25–27). In a large observational cohort study of 19,213 women with a history of miscarriage or infertility trying for a pregnancy, TPOAb was found in 9.5% of asymptomatic women (28). TAI was found in 5.9% of women at the first intra-uterine insemination and in 25% of women undergoing ART (17, 29). The secondary analysis of data from two multicenter, randomized, controlled trials reported that 8.6% infertile women had TPOAb (30). In one study not showing significant difference of TAI prevalence between the whole infertile population and the controls, higher prevalence was observed in women with female causes and, the highest, in women with endometriosis (29%) (31). This association was not confirmed in a subsequent study showing TPOAb in 14.9% of women with endometriosis and in 22.2% of the control group (32). An increased prevalence of TAI has also been reported in women with polycystic ovary syndrome (PCOS). In a prospective study TPOAb/TgAb or hypoechoic pattern at thyroid ultrasound was significantly higher in PCOS women compared to controls (26.9 vs. 8.3% and 42.3% vs. 6.5% respectively). PCOS patients had a higher mean TSH level and a higher incidence of TSH levels above the upper limit of normal (22). Again these findings were not confirmed in a recent study on 210 women with PCOS and 343 age matched controls: no differences were found in the prevalence of TPOAb and/or hypohecoic pattern at thyroid ultrasound between patient and controls (4.8% and 7.6% and 9.3% and 12.3%, respectively) but subjects with TAI showed significantly higher adiposity and insulin resistance index than those without (33). Finally, in a meta-analysis pooling four studies, TAI was more prevalent in euthyroid women with idiopathic infertility with an odds ratio of 1.5 (34). It is worth remembering that in most of the cited studies TAI is defined based on the presence of TPOAb. In a prospective study both TPOAb and TgAb were present in 8% of the cases but isolated TgAb prevalence was close to that of isolated TPOAb (4% vs. 5%). Interestingly women with TgAb had significantly higher serum TSH levels compared with women without TAI. The study also showed a higher prevalence of TAI in infertile patients (35). In 436 women attending a fertility center, TPOAb and TgAb were detected in 10.6% and 9.2%, respectively, overlap was found in 4.6% (36). Therefore, up to 5% of TAI women can be missed if only TPOAb is measured. On the other hand, it is well known that circulating thyroid autoantibodies could wane in pregnancy or be absent, outside pregnancy, in the so-called “seronegative chronic autoimmune thyroiditis” (37). **Table 1** summarizes some of the studies on the prevalence of TAI in infertile women.

**Table 1:** Prevalence of thyroid autoimmunity in infertile women.

Author, year	Patients’ characteristics	Hallmark of TAI	Percentage of TAI+		Conclusion
Karakan, 2013 (25)	253 women undergoing ART for male/female causes of infertility	TPOAb, TgAb	13.4%		No difference in causes of infertility between TAI+ and TAI-
Dhillon-Smith, 2020 (28)	19,350 women, miscarriage/infertility	TPOAb	9.5%		Higher prevalence of TAI in obese women
Hamad, 2021 (29)	584 women undergoing ART for female/male/combined infertility	TPOAb, TgAb	25.3%		TAI more prevalent in women with combined infertility factors
			** Percentage of TAI+**		
			**Patients**	**Controls**	
Poppe, 2002 (31)	438 women with various causes of infertility, 100 age-matched healthy parous controls	TPOAb	14%	8%	Higher prevalence of TAI in women with female causes (the highest in endometriosis)
Petta, 2007 (32)	148 women with endometriosis 158 without	TPOAb, TgAb	14.9%	22.2%	No difference in TAI prevalence
Janssen, 2004 (22)	175 patients with PCOS, 168 age-matched without PCOS	TPOAb, TgAb	26%.	8.3%	Threefold higher prevalence of TAI in patients with PCOS
		Hypoechoic pattern at T-US	42.3%	6.5%	
Kim, 2020 (33)	210 women with PCOS, 343 age-matched controls	TPOAb	4.8%	7.6%	No difference in TAI prevalence
		Hypoechoic pattern at T-US	9.3%	12.3%	
Unuane, 2013 (35)	356 women, female infertility, 458 not consulting for infertility/male infertility	TPOAb, TgAb	19%	13%	Higher prevalence of TAI in infertile women

TAI, thyroid autoimmunity; ART, assisted reproductive technology; TPOAb, thyroperoxidase antibody; TgAb, thyroglobulin antibody; PCOS, polycystic ovary syndrome; T-US, thyroid ultrasound.

In conclusion, a higher prevalence of TAI cannot be demonstrated in all infertile women, but it is plausible in women with PCOS and with unexplained infertility. In the case of PCOS TAI might further affect fertility, being also associated to higher TSH levels and to a worse metabolic phenotype.

## Pathophysiology

A clear pathophysiological link connecting TAI to infertility and to pregnancy outcome after spontaneous conception or ART has not been identified. There are several hypotheses and potential points of action have been proposed. Thyroid hormone-dependent as well as thyroid hormone-independent immunological effect of TAI on the ovary, on the uterus, and on fetoplacental unit have all been implicated. Moreover, TAI could represent a peripheral marker of a general immune imbalance affecting fertilization, implantation, and pregnancy maintenance (15–17). The most relevant hypotheses are briefly illustrated.

### Thyroid Antibodies Are Directly Pathogenic to the Reproductive Organs

In searching a potential target of TAI in infertility and ART outcome, antithyroid antibodies direct binding and damage to the reproductive organs has been investigated. Interestingly it has been demonstrated that antithyroid antibodies can pass through the blood–follicle barrier during the maturation period. In a study of 31 women undergoing IVF, TPOAb and TgAb were measurable in the follicular fluid, on the day of oocyte retrieval, in 14 patients with TAI and in none of the negative control. The follicular fluid concentrations of antithyroid antibodies were approximately half with respect to those in the serum thus indicating that they pass the blood-follicle barrier and reach concentration proportional to their blood levels. In this study oocyte fertilization, good quality embryos, and pregnancy rates were lower in women with TAI than in negative controls, while early miscarriage rate was higher. This “follicle hypothesis” suggests that presence of antithyroid antibodies may create a cytotoxic environment that damages the maturing oocyte reducing its quality and fertilization potential (38). A recent study confirmed the association of antithyroid antibodies in follicular fluid and ART outcome. In 52 women undergoing ART a statistically significant correlation was found between the levels of TPOAb and TgAb in serum and follicular fluid. Pregnancy rates per initiated cycle and per embryo transfer cycle were lower in TAI women thus suggesting a negative effect on the post-implantation embryo development (39). Although only in three patients, thyroid peroxidase has been demonstrated, by immunocytochemistry, in the granulosa cumulus cells of the human ovarian follicle, thereby supporting the hypothesis that TPOAb could target their antigen directly at the level of the ovary (40). In an autoimmune thyroiditis animal model antithyroid antibodies were evidenced on the surface of pre-implantation embryos (41). Also, it has been shown that human anti-zona pellucida antibodies recognize antigens within the murine thyroid tissue. It can be speculated that the zona pellucida, which has an important functional role in the interaction between the oocyte and the sperm cell and in pre-implantation, may be a target for antithyroid antibodies (42). Although the evidence is limited, a role of a hostile immune environment at the level of the ovary, with TPO as the direct antigen, has been proposed as one of the non-thyroid hormone dependent mechanisms at least in the early stage of autoimmune process in infertility (16). These antibodies may generate an inflammatory response that alters the milieu of the maturing oocyte affecting ovarian reserve and embryo quality.

### Thyroid Autoantibodies Are an Epiphenomenon of a Generalized Immune Dysfunction

An alternative pathogenetic hypothesis considers that TAI might be a marker of both generalized humoral and cellular immune dysfunction. Polyclonal lymphocyte B activation is more frequent in TAI and is associated with the increased levels of non-organ-specific antibodies, such as antinuclear antibodies (ANA), anti-dsDNA, anti-ssDNA, and antiphospholipid antibodies (aPL). Indeed, most of these autoantibodies are associated with reproductive failure; aPL can cross-react with trophoblast–placental tissue thus reducing trophoblast viability, syncytialization and invasion (43). An increased rate of recurrent miscarriage is reported in patients with poly-autoimmune disorders as compared with patients affected by isolated TAI (44). An increased levels of antibodies against laminin-1 (LN-1), that have been associated with reproductive failure, have been demonstrated in serum and follicular fluid of infertile women with TAI. The serum levels of these antibodies were inversely correlated with oocyte count, along with a significantly reduced implantation rate and pregnancy rate (45). The concurrent presence of anti-ovarian autoantibodies, hallmarks of premature ovarian insufficiency (POF) obviously can affect the immune homeostasis of oocytes and impair ovarian reserve. POF can occur in isolation but is often associated with other autoimmune conditions. Hypothyroidism, TAI, and Graves’ disease are the most seen associated disorders (46). Lymphocyte T cells, T helper (Th)1/Th2 balance, and regulatory T (Treg) cells play an important role in pregnancy; an altered cellular immune status and increased secretion of inflammatory cytokines is considered to contribute to adverse pregnancy outcome such as miscarriage and pre-term birth (PBT) (47). IL-2 and INF-γ, produced by Th1 cells, play important roles for the induction of implantation failure and abortion while the proinflammatory cytokine IL-17 is involved in the pathogenesis of abortion and PTB (48). Animal models have shown that an increased incidence of fetal loss and enhanced Th1 cell proliferation in Tg-immunized mice (49). Also natural killer (NK) cells dysregulation in the peripheral blood, with a prevalence of cytotoxic over immunoregulatory is closely related to reproductive failures, including recurrent miscarriage (RM) and infertility (50). Indeed, phenotypic and functional analysis on peripheral blood mononuclear cells from healthy donors and from TAI patients showed Th1 oriented changes of innate immunity, elevated NK, and NKT-like cells ratios, and enhanced natural cytotoxicity in TAI positive euthyroid women (51). Proinflammatory cytokine such as IL-2 and IL-17 as well as interferon gamma have been shown increased in the serum and/or in the follicular fluid of patients with TAI along with a quantitative and qualitative changes in endometrial T cells with reduction of secretion of IL-4 and IL 10 (52–54). Also, an increase in the percentage of cytotoxic NK cells in women with TAI was associated with reproductive failures (51). These data, although merely descriptive, suggest that cellular immune dysfunction, with a proinflammatory Th1 immune response and excessive activation of NK cells and NKT-like cells might induce the occurrence of infertility, miscarriage, and PTB in women with TAI (15).

### Thyroid Antibodies Cause a Relative Hypothyroidism

The natural history of chronic autoimmune thyroiditis is a progressive decline in the functional capacity of the thyroid gland, leading to subclinical and, ultimately, to overt hypothyroidism. A decreased functional capacity of the thyroid gland may become apparent during early pregnancy since this condition constitutes a state of extra demand of thyroid hormone production. Indeed, it is well known that TAI increases the risk of developing subclinical or overt hypothyroidism during pregnancy. In the first half of spontaneous pregnancy women with TPOAb had an increased prevalence of subclinical and overt hypothyroidism compared to controls (20.1 vs. 2.4% and 3.3 vs. 0.1%, respectively) (55). In women undergoing ART the ovarian stimulation anticipates the extra demand of thyroid hormone, which occurs only after conception during spontaneous pregnancy, and therefore TSH levels can increase significantly above 2.5 mIU/L before pregnancy in the presence of TAI (8). In early pregnancy the human chorionic gonadotropin (hCG), through its high homology with TSH, stimulates the thyroid, thus helping to maximize thyroid hormone secretion (6). An inappropriate thyroidal response to hCG stimulation has been considered an early marker of abnormal thyroid functional capacity in women with TAI. A study gathering data from two large population-based prospective cohorts showed that there was a positive association of hCG with FT4 as well as a negative association of hCG with TSH in TPOAb-negative women but not in TPOAb-positive women during early pregnancy. Women with TPOAb and lower FT4 than expected had a higher risk of premature delivery. This study therefore links the adverse pregnancy outcome to changes in thyroid function in TAI (56). It is believed that women with TAI may not benefit from the stimulation due to hCG aimed to meet the increased demand of thyroid hormones in early pregnancy. A recent study showed that TgAb also interferes with the thyroidal response to hCG. In 822 women at 7-20 weeks of gestation, when TSH was within the pregnancy-specific reference range, high concentrations of TPOAb and TgAb attenuated the FT4 stimulation and TSH suppression induced by hCG. This effect was more pronounced when both TPOAb and TgAb were present (57). Whether these data can be extrapolated for the dose of hCG used for the ovulation induction remains unknown and deserves further investigation (11). The antithyroid antibodies-induced impaired response of thyroid to hCG is not in contrast with their action on the ovary. Indeed, a two-stage mechanism has been recently proposed: at early stages of autoimmunity, when TPOAb levels are low and thyroid function is normal, the main effect of the antibodies is the creation of a hostile immune environment at the level of the ovary; as the autoimmune process progresses, the impaired response to hCG also becomes apparent (16).

## Thyroid Autoimmunity and Assisted Reproductive Technology Outcome

Several studies addressed the effect of TAI on pregnancy outcomes in euthyroid women who had conceived spontaneously (58). Euthyroid women with TAI carried twofold risk of miscarriage and were found to have slightly higher age and TSH levels (59). A triple or double risk of miscarriage or preterm birth, respectively, has also been reported in women with TPOAb (60). In a recent study the presence of TPOAb was also associated with preterm birth; the association did not appear to be related to differences in thyroid function, although the highest risk of preterm birth was observed in women with TPOAb and TSH >4.0 mIU/L. No association of TgAb positivity with preterm birth was found (61). In infertile women the presence of TPOAb increased the risk of miscarriage while a preconceptional TSH ≥ 2.5 mIU/L did not (30). Likewise, there has been intense interest in whether TAI may affect the success of the infertility treatments. Whenever possible primary approaches to female infertility are targeted to the identified cause and include ovulation induction for women with ovulatory dysfunction; endoscopic or surgical procedures to treat tubal obstruction or endometriosis. Intrauterine insemination (IUI) with donor or partner sperm is the first line treatment in couples with unexplained infertility, cervical factor infertility, and male infertility. When all these approaches fail ART is indicated. ART comprises both *in vitro* fertilization (IVF) and intracytoplasmic sperm injection (ICSI). Although the two main indicators of ART success are the proportion of clinically pregnant women and the proportion of miscarried women, there are several parameters used to evaluate each step of the *in vitro* and *in vivo* ART outcomes starting from the ovarian response to the controlled hyperstimulation (COH), and the number of oocytes retrieved (NOR) and going further to the fertilization rate (FR) and to the implantation rate (IR). The most relevant outcome is the pregnancy rate (PR), and, better, the clinical pregnancy rate (CPR). Pregnancy indeed can be defined as either biochemical (i.e., a transient rise in hCG concentration) or clinical (the formation of a gestational sac and fetal heartbeat) and the outcome can be defined as live birth rate (LBR), ending with a miscarriage, miscarriage rate (MR), or with a pre-term birth (PTBR). Also, infant/neonatal complications are of interest, among these, above all, low birth weight (LBW).

### Ovarian Reserve in Women With Thyroid Autoimmunity

The term ovarian reserve (OR), that is, the complete follicle pool and, mostly, the functional ovarian reserve (the number of maturing growing follicles after recruitment), expresses the woman’s reproductive potential. Several studies addressed the effect of TAI on OR by evaluating its predictive markers such as high basal follicle stimulating hormone (FSH) levels, antral follicle count (AFC), and anti-Müllerian hormone concentrations (AMH). In 30 adolescent patients with TAI, AMH levels were comparable to controls. Serum AMH was negatively correlated with TSH but not with TPOAb or TgAb levels (62). Also in a case-control study 30 adolescent girls, newly diagnosed with TAI, had normal ovarian reserve based on measurements of AMH, inhibin B, FSH, LH/FSH ratio, estradiol, and AFC (63). In 775 reproductive age women, without any thyroid or ovarian dysfunction, those with AMH levels in the lower quartile for age had higher levels of TPOAb at baseline while there was no statistically significant difference in thyroid hormones compared to women with AMH in the higher quartiles. Over a 12-year follow up FT4 was decreased in all quartiles whereas TPOAb increased only in women with AMH in the low quartile (64). In a study involving 108 euthyroid TAI patients with regular menstrual cycles, either treated or not with LT4, lower AMH levels were found in patients compared to the age-matched healthy women (65). OR is one of the most important determinants of downstream ART outcomes since it can affect the ovarian response to the COH protocols. Indeed, in 288 infertile women (55 euthyroid with TAI and 233 without) undergoing their first ART, higher levels of AMH were associated with better COH outcome as reflected by the estradiol/recombinant FSH ratio (E2/rFSH) and by the number of oocytes reaching metaphase II (M II oocytes). Women with TAI had lower AMH and higher FSH levels compared to TAI negative, but a poorer COH outcome was observed also in women with TAI and higher AMH levels. Thus, although diminished OR impairs COH outcome independently from TAI, the latter reduces ovarian response in women with preserved OR (66). The same group had previously also shown the effect of thyroid function on the above-mentioned markers of COH. In 262 women undergoing ART, those with TAI and TSH levels below 2.5 mIU/L had better ovarian response demonstrated by higher serum estradiol levels, higher E2/rFSH ratio, and a greater number of M II oocytes compared with those with TSH >2.5 mIU/L (67). In a study involving 1044 infertile women eligible for IUI/IVF, TSH levels, TPOAb positivity, and TgAb positivity were comparable between patients with variable ovarian reserve according to AMH levels. However, after patients with known causes of diminished ovarian reserve (i.e., genetic) were excluded, TPOAb positivity was higher in patients with low ovarian reserve thus indicating an association of TPOAb with idiopathic low ovarian reserve in unexplained infertility (68). In a study group of 436 women seeking fertility treatment, thyroid function or TPOAb positivity was not associated with AFC, while TgAb positivity was associated with a higher AFC. However, among women with diminished ovarian reserve or unexplained infertility, TPOAb and not TgAb, as well as lower FT3, were associated with a lower AFC (36). On the contrary in a large study including 5000 women no differences were found in the prevalence of TAI as well as of overt or subclinical hypothyroidism in women with low, normal, or high ovarian reserve expressed by age-specific AMH values (69). Also in 225 infertile women TSH <3.0 mIU/L was associated with significantly higher AMH compared to those with TSH ≥3.0 mIU/L after adjustment for thyroid autoimmunity and age, thus suggesting that thyroid function, even in the euthyroid range, has a more significant impact than TAI in the ovarian reserve (70).

### *In Vitro* and *In Vivo* ART Outcome in Women With Thyroid Autoimmunity

Multiple clinical studies, systematic reviews, and metanalyses have evaluated the impact of TAI in ART *in vitro* and *in vivo* outcomes with conflicting conclusions. Indeed, the studies differ for sample size, design, causes of infertility, and type of ART, TSH level cut-off used to define euthyroidism as well as for the endpoints. Embryo quality was assessed in 431 embryos in euthyroid women with low ovarian reserve undergoing IVF. Comparable embryo quality was observed between women with TSH low-normal or high normal TSH (cut off 2.5 mIU/L) but impaired embryo quality was observed in women with TPOAb and TSH ≤ 2.5 mIU/L. Increasing TSH affected embryo quality and a trend toward the same effect was observed in women with TPOAb (71). Also, in a retrospective cohort study of 123 euthyroid women with or without TAI undergoing ART, embryo quality was significantly impaired in women with at least one autoantibody while no differences were found in AMH, FSH, and in the number of oocytes picked up and fertilized as well as in implantation rate and in pregnancy rate (72). Several prospective cohort studies releveled lower LBR and increased MR in euthyroid women with TPOAb undergoing IVF/ICSI. In 234 euthyroid women undergoing the first cycle of ART no differences in pregnancy rate were observed but the TAI women had threefold higher risk of miscarriage (73). No differences in pregnancy rate were observed between 412 TPOAb negative women and the 72 TPOAb positive that had been randomly assigned to receive LT4 or placebo. Nevertheless, a twofold risk of miscarriage was observed in euthyroid TPOAb positive women not subjected to LT4 treatment. The risk of miscarriage was not reduced by LT4 treatment in TPOAb positive women. This links the miscarriage to an abnormal immune response rather than to subsequent mild thyroid failure (74). Also, in a prospective study involving 590 infertile hypothyroid women treated with LT4, to maintain TSH levels below 2.5 μIU/mL, higher TPOAb titer was one of the risk factors for miscarriage, after IUI and IVF despite appropriate treatment (75). Among 194 euthyroid women undergoing IVF, the 60 with TPOAb/Tg Ab showed lower clinical pregnancy and live birth rate when confronted with controls. The same parameters were improved in 30 antithyroid antibodies-positive women treated with prednisone (76). On the contrary no effect on pregnancy outcome was observed in several retrospective studies published in the last 20 years. In 416 euthyroid women no differences in pregnancy and delivery rates were observed between women with and without TPOAb. However, women with TPOAb who failed to become pregnant or miscarried had high-normal TSH compared to the ones who delivered and compared to women without TPOAb (77). In a study enrolling 2406 women, analysis of cumulative delivery did not show differences between TAI positive and negative women after 6 IVF/ICSI cycles (78). Also, in more recent studies no impact of TPOAb and TgAb was observed in the MR and on the *in vitro* ART outcome such as NOR, FR, and embryo quality (26, 79, 80). In 584 women undergoing IVF/ICSI with TSH between 0.45 and 4.5 μIU/ml, NOR, FR, and CPR did not significantly differ between TAI positive and TAI negative. Subgroup analysis for only primary infertility patients showed a statistically significant lower CPR in TAI positive compared to TAI negative (29).

Also looking at meta-analysis studies conflicting results are reported. A meta-analysis of prospective studies reporting data on 1098 subfertile women undergoing IVF reported high miscarriage risk in euthyroid TAI women undergoing IVF but did not find significant difference in FR, CPR, and delivery rates (81). Comparable results were reported in a further study showing that TAI increased the risk of MR while it did not affect NOR, implantation, and clinical pregnancy rate. The effect persisted after meta-regression analysis of age and TSH serum levels although the authors do not exclude possible modifying effects of these two variables (82). A more recent metanalysis including 14 studies performed on euthyroid TAI women did not show differences in *in vitro* and *in vivo* ART outcomes (CPR, MR, LBR per cycle, number of embryos transferred, NOR) neither for maternal age nor TSH levels when compared to women without TAI (83). Thus, heterogeneous results are reported and, also, the risk for adverse outcome is reported lower in the more recent metanalysis studies than in the previous ones (11). The explanation for the heterogeneity of these results can be found in the study design, in the TSH threshold used to define euthyroidism and as well as in the type of ART treatment and ovulation induction protocols. The effect of preconception TSH levels in the ART outcomes has been the subject of a recent meta-analysis. The study reported that when the TSH cut-off value for SCH was set at 2.5 mIU/L, no significant differences were observed in ART-related outcomes between SCH patients and normal women. On the contrary there was an increased risk of MR in women with SCH when a TSH cut-off value of 3.5–5.0 mIU/L was used (84). Regarding the studies on ART procedure, some have shown that the ICSI outcome was not affected by TAI. Indeed, in the last years ICSI has become the most popular insemination method worldwide. In a meta-analysis of studies including 1855 ICSI cycles, women with and without TAI, not differing for age, had comparable CPR, MR, and LBR. The authors propose that the presence of TAI may become a new indication for ICSI, independently of the cause of infertility, as it may overcome the detrimental impact of autoimmunity on fertilization and embryo quality (85). Indeed, previous observations reported that ICSI, which requires no interaction between the sperm cell and the zona pellucida, resulted in similar PR in women with or without TAI undergoing ART for male infertility (86). If this is true different outcomes are expected between IVF/ICSI and IUI. In a retrospective cohort study, enrolling 3143 patients, no significant different outcomes (PR, MR, LBR) were observed after IUI in euthyroid women with and without TAI also when comparing subgroups according to TSH level (TSH ≥2.5 mIU/L vs. TSH <2.5 mIU/L) (87). This latter finding was confirmed in a study enrolling 726 euthyroid women undergoing IUI for unexplained infertility. In this study, cycle characteristics and pregnancy outcomes (CPR, MR, LBR) of patients with serum TSH levels between 0.3-2.5 mIU/L and 2.5-4.5 mIU/L were compared and no statistically significant differences could be detected (88). In conclusion, although ART is an ideal model to analyze the effect of TAI on each stage of the reproductive process, compared to spontaneous conception, the multitude of variables present in the studies (age, causes of infertility, protocols for ovarian stimulation, the type of ART treatment, thyroid antibody levels, thyroid function) make it difficult to attribute to TAI per se a role on adverse outcomes and also to identify the TSH level threshold at which a negative impact on ART is expected, keeping in mind that TSH above 2.5 mIU/L is easily encountered after ovarian stimulation and even more so in the presence of TAI (78, 84). In **Table 2** are illustrated the above-mentioned studies addressing the effect of TAI on the prediction markers of ovarian reserve/response as well as on the pregnancy outcome.

**Table 2:** Studies addressing the effect of TAI on ovarian reserve/response and on assisted reproductive technology (ART) outcome.

Author, year	Patients’ characteristics	Study outcomes	Thyroid function	Main conclusion
Polyzos, 2015 (69)	5000 women, infertility work up/other reasons	TAI prevalence in women with variable OR	EU/SCH/OH	No difference of TAI in women with variable OR
Chen, 2017 (68)	1044 women eligible for IUI/IVF	TAI prevalence in women with variable OR	EU	Idiopathic low OR associated with TAI
Korevar, 2018 (36)	436 women (46 TAI+) infertility work up	Association of TAI with OR	EU	TAI associated with lower AFC in women with unexplained infertility or diminished OR
Weghofer, 2016 (70)	225 women (25 TAI+) infertility work up	Association of TAI/TSH with OR	EU	TSH <3.0 mIU/L associated with higher OR
Magri, 2015 (66)	288 women (55 TAI+), IVF	Ovarian response to COH	EU	Poorer ovarian response in TAI
Weghofer, 2016 (70)	98 women (17 TAI+) with low OR, IVF	EQ	EU	Poorer embryo quality in TAI
Andrisani, 2018 (72)	123 (29 TAI+), IVF/ICSI	EQ, IR, PR	EU	Poorer EQ in TAI, no differences in IR and PR
Poppe, 2003 (73)	234 women (32 TAI+), IVF/ICSI	LBR, CPR, MR	EU	No difference in CPR in TAI, higher MR in TAI
Negro, 2005 (74)	484 (72 TAI+), IVF/ICSI	PR, MR	EU	No difference in PR, higher MR in TAI
Negro, 2007 (77)	416women (42 TAI+), IVF/ICSI	NOR, LBR, CPR, MR	EU	No effect on CPR
Tan, 2014 (86)	835 women (110 TAI+), ICSI	CPR, MR, PTB	EU	No effect of TAI
Litwicka, 2015 (76)	194 (60 TAI+), IVF	NOR, LBR, CPR, MR,	EU	Lower LBR, higher MR in TAI
Lukasuk, 2015 (26)	573 (114 TAI+), ICSI	NOR, LBR, PR, MR,	EU	No effect on PR, LBR, MR, lower NOR in TAI
Unuane, 2016 (78)	2406 women (33 TAI+), ICSI	LBR	EU	No effect of TAI
Unuane, 2017 (87)	3143 women (187 TAI+), IUI	LBR, CPR, MR	EU	No effect of TAI
Poppe, 2020 (79)	279 women, ART, IVF/ICSI	NOR, FR, EQ	EU/SCH	No effect of TAI/SCH
Hamad, 2021 (29)	584 women (148 TAI+), IVF/ICSI	CPR	EU	No effect of TAI

TAI, thyroid autoimmunity; OR, ovarian reserve; AFC, antral follicle count; EQ, embryo quality; NOR, number of oocytes retrieved; IR, implantation rate; CPR, clinical pregnancy rate; LBR, live birth rate; MR, miscarriage rate; EU, euthyroidism; SCH, subclinical hypothyroidism; IVF, in vitro fertilization; ICSI, intracytoplasmic sperm injection.

## Implications For Screening and Management in Clinical Practice

Universal thyroid screening in pregnancy fulfills most criteria for a beneficial and cost-effective screening program, aimed to reduce fetal and maternal complications, but it is still a matter of debate (89). Despite the discordant findings of the studies regarding the association of antithyroid antibodies with infertility and adverse outcome after ART, infertile women constitute a selected group of patients for whom specific recommendations for screening and management come from the guidelines. According to ATA, women with infertility or with a history of miscarriage are “at high risk” of developing thyroid dysfunction and for them serum TSH concentration is recommended as soon pregnancy is confirmed with reflex TPOAb measurement if TSH is >2.5-10 mIU/L. Women undergoing ART, instead, fulfill the criteria for TSH universal screening (13). As per the very recent ETA guidelines, specifically referring to ART, TSH and TPOAb should be measured in women seeking medical advice for infertility; “the TgAb can be added systematically according to the local regulatory authority rules” while it should be measured when TSH levels are higher than 2.5 mIU/L and TPOAb are absent (14). Thyroid ultrasound, which is useful to calculate the gland volume as well as to detect features compatible with autoimmune thyroiditis, is not mentioned in ATA guidelines while is recommended by ETA if TSH is >2.5 mIU/L and TPOAb is negative. Regarding the treatment, there is not debate about the need to start LT4 in women with overt hypothyroidism who undergo ART or become pregnant spontaneously independently from their thyroid antibodies status. For ATA the decision to treat women with TSH >2.5 and 10 mIU/L is based on TSH level and on the TPOAb status. Treatment is recommended in TPOAb-positive women when TSH is above 4 mUI/mL (if trimester specific ranges are not available) while it can be considered in TPOAb positive women with TSH >2.5 < 4 mUI/L and in TPOAb negative women with TSH >4 < 10 mUI/L. According to ATA, for euthyroid women undergoing ART LT4 treatment, at low starting dose of 25-50 μg “may be considered given its potential benefits in comparison to its minimal risk”. Indeed the 2.5 mUI/L TSH threshold still has value in women undergoing ART since ATA states that it is prudent to recommend treatment for “any TSH elevation over 2.5 mUI/L” before the procedure. On the contrary, also in case of ART, for some authors, a TSH level of 4 mIU/L is the cut-off for treatment since LT4 seems to increase LBR only when TSH is >4.0 mIU/L and also taking into account potential harmful effects of overtreatment (17, 90). The ETA guidelines recommend that women with serum TSH >4.0 mIU/L planning ART should be treated with LT4 independently of their TPOAb status, **Table 3**. In women with TAI and TSH levels >2.5<4 mIU/L, LT4 treatment at low starting dose of 25-50 μg LT4, before ovarian stimulation, could be “initiated in a case-by-case manner” such as in women with recurrent miscarriage, in women over 35 years of age, and with ovarian causes of infertility (14). According to ETA, LT4 treatment of TAI women with TSH levels >2.5<4 mIU/L could optimize ovarian reserve and improve embryo quality while there is no definitive evidence of benefits on pregnancy outcome. Several studies have addressed the benefit of LT4 on ART outcome in euthyroid women with TAI. In one randomized clinical trial (RCT) previously mentioned, pregnancy rate was not affected either by presence of TPOAb or by treatment with LT4. Euthyroid women (TSH ≤ 2.5 mIU/L) with TAI had high MR but LT4 treatment did not improve the delivery rate (74). These findings were supported by a recent large RCT. Treatment with LT4, 25-μg/d or 50-μg/d according to the TSH ≤2.5 or >2.5 mIU/L, respectively, did not reduce MR or increase CPR and LBR among 600 Chinese euthyroid women with TAI undergoing IVF. The study excluded women at high risk of miscarriage, and women positive for antinuclear and anticardiolipin antibodies, or lupus anticoagulant to eliminate other autoimmune confounding factors. Moreover, although in a limited number of patients, no benefits of therapy were observed in women with TSH>4 mIU/L (91). This study supports the conservative approach expressed by the recent guidelines (14). Nevertheless, in a meta-analysis, also including some studies, LT4 treatment had no significant impact on live births in women with SCH or TAI undergoing IVF; however, it decreased miscarriage rates. It is worth remembering that in some studies patients characterized by TPO-positivity and TSH levels 4.0 or 4.5 mIU/L were mixed in data synthesis (92). The TABLET study, a randomized, placebo-controlled trial, investigated the effect of a fixed dose of LT4 50 μg daily, on LBR in TPOAb positive euthyroid women. This very large study included women with a history of recurrent miscarriage or receiving treatment for infertility. Prespecified subgroup analyses were completed for the primary outcome according to maternal age, the baseline TSH level (≤2.5 mIU/L or >2.5 mIU/L), and infertility treatment. LT4 treatment did not result in a higher LBR compared with placebo (93). The studies not showing a benefit of LT4 support the hypothesis that the increased risk of miscarriage in TAI is linked to an abnormal immune response rather than to mild thyroid failure. In this regard ETA guidelines suggest ICSI as the preferred fertilization method in women with TAI to overcome potential negative effects of antithyroid antibodies on oocytes and embryos (14). Currently no treatments have been shown to reduce thyroid autoimmunity and this aspect continues to be studied (94). In a previous study the use of glucocorticoids demonstrated an increase in pregnancy rates with treatment compared with placebo (76). In a more recent study it has been demonstrated that glucocorticoid may improve the pregnancy outcomes of ART women with antithyroid antibodies positive, but does not reduce the risk of miscarriage (95). Selenium supplementation decreases thyroid autoantibodies concentration during pregnancy but with no benefit on fetal or maternal outcomes (96).

**Table 3:** Recommendations for women with infertility and/or undergoing assisted reproductive technology (ART) according to the ETA 2021 guidelines (14).

Test/Treatment	ETA 2021
TSH screening	Women seeking care for infertility
Recommended TSH upper limits	4.0 mIU/L or ULRR*
TPOAb measurement	Women seeking care for infertility
TgAb measurement	If TSH>2.5 mIU/L and TPOAb negative
Thyroid ultrasound	If TSH>2.5 mIU/L and TPOAb negative
L-T4 treatment in women undergoing ART	Recommended in overt hypothyroidism Recommended if TSH is >4 mIU/L with/without TAI Suggested in TAI if TSH is >2.5< 4 mIU/L on a case-by-case basis
Fertilization preferred method in TAI	ICSI suggested

TSH, thyroid stimulating hormone; ART, assisted reproductive technology; ULRR, Upper limit reference range. TAI, thyroid autoimmunity. TPOAb, thyroperoxidase antibody; TgAb, thyroglobulin antibody; L-T4, levothyroxine; OS, ovarian stimulation ICSI, intracytoplasmic sperm injection. ART, assisted reproductive technology.

*If ULRR is >4.0 mUI/L.

## Conclusions

Overt thyroid dysfunction leads to menstrual disturbances, fertility problems, and pregnancy complications. Also, thyroid autoimmunity, even in the setting of euthyroidism, might adversely affect fertility and reproductive outcome by creating a cytotoxic environment that damages the maturing oocyte reducing its quality and fertilization potential and by inducing a subtle dysfunction during early pregnancy. Thyroid autoimmunity is associated with some causes of infertility (ovarian and idiopathic) and with some adverse pregnancy outcome, such as miscarriage, after assisted conception. Women with TAI undergoing ART are an ideal model to analyze the effect of thyroid autoimmunity/dysfunction on each stage of the reproductive process, compared to spontaneous conception. A better understanding of the pathophysiology may have an impact on the therapeutic approach. Although many questions remain unanswered, there is no doubt that it is justified for women seeking medical advice for infertility screening for thyroid dysfunction and autoimmunity. The decision for treatment should be based on the current evidence and recommendations but it cannot omit an individualized clinical judgement on the cause of the infertility as well as on the obstetric history of the women.

## Author Contributions

IB: substantial contributions to the conception and design of the work; reviewing the literature; drafting the work; final approval of the version to be published; and agreement to be accountable for all aspects of the work in ensuring that questions related to the accuracy or integrity of any part of the work are appropriately investigated and resolved. CG, GDD, GF, and GN: contributions to the design of the work; revising the work critically for important intellectual content; final approval of the version to be published; and agreement to be accountable for all aspects of the work in ensuring that questions related to the accuracy or integrity of any part of the work are appropriately investigated and resolved. All authors contributed to the article and approved the submitted version.

## Conflict of Interest

The authors declare that the research was conducted in the absence of any commercial or financial relationships that could be construed as a potential conflict of interest.

## Publisher’s Note

All claims expressed in this article are solely those of the authors and do not necessarily represent those of their affiliated organizations, or those of the publisher, the editors and the reviewers. Any product that may be evaluated in this article, or claim that may be made by its manufacturer, is not guaranteed or endorsed by the publisher.
